# Exercise therapy in the application of sleep disorders

**DOI:** 10.3389/fneur.2024.1324112

**Published:** 2024-06-19

**Authors:** Yuhang Zhao, Qiang Dai, Yuhao Li, Chenyang Li

**Affiliations:** ^1^School of Kinesiology and Health, Capital University of Physical Education and Sports, Beijing, China; ^2^Graduate School, Pai Chai University, Daejeon, Republic of Korea; ^3^Graduate School, Dankook University, Yongin, Republic of Korea

**Keywords:** neurological injuries, sleep disorders, exercise therapy, molecular mechanisms, sleep–wake regulation

## Abstract

Sleep disorders often accompany neurological injuries, significantly impacting patient recovery and quality of life.The efficacy and adherence of traditional treatment methods have certain limitations. Exercise has been found to be a highly beneficial treatment method, capable of preventing and alleviating neurological injuries and sleep disorders. This article reviews relevant research findings from both domestic and international sources over the past few decades, systematically summarizing and analyzing the application of exercise therapy in sleep disorders,strategy of exercise intervention program and the potential molecular mechanisms by which exercise therapy improves sleep disorders. Shortcomings in current research and suggestions are presented, providing a reference for future in-depth studies on exercise interventions for sleep disorders.

## 1 Introduction

Sleep disorders are brain diseases that have long-term negative impacts on both physical and mental health (1). Sleep disorders are closely related to neurodegenerative diseases. Among patients with multiple sclerosis, Parkinson’s disease, Alzheimer’s disease, stroke, traumatic brain injury, or epilepsy, the prevalence of sleep disorders varies from 25 to 60% (2). Structural and functional abnormalities in patients’ brain tissues involve multiple brain regions, primarily including neural pathways related to memory, emotional processing, and the generation and regulation of sleep (3). Animal experiments have shown that sleep deprivation in animals can lead to abnormal changes in the transcription and translation of various genes, including those related to mitochondrial energy metabolism, endoplasmic reticulum, and synapse-related genes. Furthermore, it was found that the initiation of apoptosis pathways in mitochondria and endoplasmic reticulum is an important mechanism of damage caused by sleep disorders (4).

Common treatments for sleep disorders primarily include medication and Cognitive Behavioral Therapy for Insomnia (CBT-I). However, 40% of patients with chronic insomnia symptoms cannot achieve long-term relief through these treatments (5). While traditional drug treatments have some effectiveness, they come with certain limitations and side effects, such as the risk of dependency and drug misuse (6, 7). CBT-I therapy is costly and highly dependent on specialized clinical practitioners, making it unsuitable as a routine treatment (8). Therefore, researchers have begun to explore new non-pharmacological interventions, with exercise therapy emerging as a safe, economical, and feasible option. Exercise therapy refers to the enhancement of an individual’s health status and quality of life through sports exercise, physical training, and other physical activities. Numerous studies have shown that exercise therapy has a positive impact on mental health, metabolic diseases, and chronic pain (9–11). Recent research has also demonstrated that exercise therapy can significantly improve sleep quality, extend sleep duration, and alleviate symptoms of sleep disorders (12–14). It is hypothesized that exercise therapy may produce its therapeutic effects by modulating neurotransmitters, hormones, and sleep regulation systems, among other molecular mechanisms.

However, current theoretical and practical research in the field of exercise therapy for sleep disorders has not yet received sufficient attention from scholars in our country, and our understanding of the specific mechanisms of action in sleep disorders remains limited. Hence, these issues require further in-depth exploration and research. Therefore, this review aims to systematically summarize and analyze the application, effects, and possible molecular mechanisms of exercise therapy in the treatment of sleep disorders. Firstly, we will introduce the definitions and classifications of sleep disorders and exercise therapy. Subsequently, we will review the therapeutic effects of exercise therapy on sleep disorders, including its impact on the symptoms of sleep disorders, sleep quality, and sleep duration, as well as its current application status in different populations. We then compared exercise therapy with traditional treatments for sleep disorders. Lastly, we will explore potential molecular mechanisms. Exercise therapy may produce its therapeutic effects by modulating neurotransmitters, neural plasticity, and neuroinflammation, among other mechanisms. Through a comprehensive analysis of existing research, we hope to provide guidance for further research and application of exercise therapy in the treatment of sleep disorders, and to offer a reference for clinical practice.

## 2 Overview of sleep disorders

### 2.1 Definition and classification of sleep disorders

Sleep disorders are characterized by a subjective experience of dissatisfaction with sleep due to frequent and persistent difficulties in falling asleep and/or maintaining sleep. As a result, the quality and duration of sleep cannot meet normal physiological needs (15). Specific features include: ① dissatisfaction with sleep quality or duration; ② at least three nights per week, for at least one month, experiencing initial sleep disorders (latency to sleep onset exceeding 30 min), middle insomnia (awakening for more than 30 min after sleep onset), or late insomnia symptoms (awakening 30 min earlier than expected with total sleep time less than 6.5 h); ③ concurrent severe distress or daytime dysfunction related to sleep difficulties, including decreased alertness, fatigue, irritability, and other symptoms (16, 17).

According to the International Classification of Sleep Disorders, Third Edition (ICSD-3), sleep disorders are categorized into eight main types: ① Insomnia disorders; ② Sleep-related breathing disorders; ③ Central disorders of hypersomnolence; ④ Circadian rhythm sleep–wake disorders; ⑤ Parasomnias; ⑥ Sleep-related movement disorders; ⑦ Independent syndromes; ⑧ Other sleep disorders.

### 2.2 Epidemiology of sleep disorders

Sleep disorders rank among the most common sleep-related issues in the general population. Approximately one-third of adults in the United States experience symptoms of sleep disorders (18). Depending on the study, the prevalence of symptoms of sleep disorders in the general adult population ranges between 30 and 50% (19, 20). This figure skyrockets to as high as 80% among individuals with psychiatric disorders (21). However, despite such high prevalence rates, only about 6–10% of adults meet the diagnostic criteria for insomnia disorder (6).

In China, sleep disorders are also prevalent. The first study to assess the overall prevalence of sleep disorders in the general Chinese population found that 15.0% of individuals suffered from these conditions (10), which is lower than in Western countries but aligns with rates observed in other Asian nations. Age and sex are the most frequently identified risk factors. The prevalence increases among elderly individuals and women. A large-scale multicentric study focusing on the elderly population in China found that sleep disorders affected as many as 38.5% of individuals aged 65 and older (22). Meanwhile, a meta-analysis on the prevalence and associated factors of sleep disorders among Chinese university students revealed a prevalence rate of 25.7% (23). Women face a 1.4 times higher risk of suffering from these disorders compared to men (24). In women aged between 40–55, there is a notably higher incidence of sleep disorders. This peak is postulated to be associated with symptoms related to perimenopause (25, 26).

### 2.3 Impact of sleep disorders on health

Poor or insufficient sleep can lead to various functional impairments in most body systems, including mental health, cognitive functions, metabolic diseases, and neurological disorders (27). Research has shown that chronic sleep disorders increase the risk of depression, hypertension, and death in the elderly (6).

A large-scale study in the U.S. involving one million patients confirmed that in the real world, sleep disorders impose a huge burden on patients. Their daytime functions are severely impaired, accompanied by multiple complications such as fatigue, dizziness, tiredness, and disorientation. They are also more likely to suffer from arterial hypertension, mental complications, anxiety, depression, or obesity (18).

Sleep disorders are a risk factor for newly diagnosed mental diseases (28), most notably depression, anxiety, and substance abuse disorders. The study suggests that patients with sleep disorders are four times more likely to develop severe depression than the general population over the next 3.5 years. The risks for anxiety disorders and substance abuse disorders are doubled and seven-fold higher, respectively (29). Sleep disorders typically have bidirectional relationships with other mental illnesses (17). Fansson-Frojmark and others (30) reported a bidirectional relationship between sleep disorders and anxiety and depression. Sleep disorders and fatigue are the most common residual symptoms in patients undergoing treatment for depression, and they increase the risk of future relapse of depression (31).

### 2.4 Mechanisms underlying sleep disorders

The exact pathophysiological mechanisms behind sleep disorders remain undetermined. However, several neurobiological and psychological models have been proposed. The primary hypotheses for the onset and persistence of insomnia are the 3P model and the hyperarousal hypothesis.

Factors influencing sleep disorders encompass behavioral, cognitive, emotional, and genetic elements (17). Conceptually, these factors are often divided into predisposing factors (Predisposing), triggering factors (Precipitating), and sustaining factors (Perpetuating) (32) - hence the cognitive-behavioral framework referred to as the 3P model. The 3P model postulates that the onset and persistence of sleep disorders result from an accumulation of the above three factors exceeding a threshold for manifestation. This model forms the foundation of the widely adopted cognitive-behavioral therapy approach to treating sleep disorders (33).

Another hypothesis is the Hyperarousal Hypothesis. The influence of circadian rhythm timing on sleep propensity prompted early suggestions that a misalignment between the circadian system timing and bedtime choices might lead to insomnia (34). The endogenous circadian pacemaker is a potent determinant of wake/sleep propensity over a 24-h period, and its misalignment can result in symptoms of insomnia, such as difficulties in sleep initiation and maintenance, and the development of circadian rhythm sleep disorders. The causal relationship might also be bidirectional; a delay in the circadian rhythm can lead to a delay in sleep onset, giving rise to conditioned insomnia or other circadian rhythm sleep disorders, further accentuating the circadian delay. Insomnias associated with early morning awakening have been linked to an earlier phase of the circadian rhythm (advanced timing) (35). Brain activity in insomnia patients shows changes consistent with hyperarousal. Individuals with insomnia may be in a state of heightened arousal, exhibiting faster brainwave frequencies during both sleep and wakefulness, increased activation of the autonomic nervous system, presenting as heart rate and heart rate variability associated with sleep, increased metabolic rates, body temperature, activity of the hypothalamic–pituitary–adrenal axis, and elevated norepinephrine secretion (36). Genetic anomalies related to sleep–wake regulation have yet to be identified in insomnia. Most studies on the role of hyperarousal in insomnia are correlational, suggesting that sleep disorders might result from insomnia, associated sleep deprivation, or co-morbidities like depression. For instance, individuals with insomnia have shown reductions in gray matter volume in the left orbitofrontal cortex and the hippocampus (37), and a higher frequency of the short allele of the 5-HTTLPR (serotonin transporter gene) (38); these markers have also been reported in depression. Therefore, further research is required to clarify the role of arousal in the pathogenesis of insomnia and its specific genetic and neurobiological mechanisms.

## 3 Sleep disorders overview of exercise therapy

### 3.1 Definition and classification of exercise therapy

Exercise is a planned, organized, repetitive, and purposeful subset of physical activity and has been recommended for the prevention and treatment of many diseases and medical conditions (39). Exercise therapy is a therapeutic method based on kinesiology, biomechanics, and neurodevelopmental science, aiming to improve physical, physiological, psychological, and mental functional disorders. It primarily utilizes force and its counterforce (40), representing the application of movement in medicine. Research consistently demonstrates that physical activity is associated with improved physical health, life satisfaction, cognitive function, and mental health. Physical activity refers to body movement that results in energy expenditure exceeding resting levels, produced by skeletal muscles.Based on the mode of exercise, exercise therapy can be divided into (41, 42):

① Aerobic exercises: Land-based training to enhance cardiopulmonary function, such as long-distance jogging, cycling, swimming, skipping rope, aerobic fitness dance, and step dance.

② Resistance exercises: Training to increase skeletal muscle strength, explosive power, endurance, and size, such as sandbags, springs, dumbbells, elastic bands, grip strengtheners, push-ups, pull-ups, squats, etc.

③ Mind–body exercises: Achieving mind–body harmony through coordinated movement and body awareness, like Pilates, yoga, tai chi, and fitness qigong.

④ Multi-modal exercises: Combining two or more types of exercise therapies.

### 3.2 Exercise therapy improves sleep health

Exercise therapy is considered a low-cost, accessible, and non-pharmacological alternative, and hence, is recommended for the prevention and treatment of sleep disorders. A majority of epidemiological studies have indicated that exercise is significantly positively correlated with better sleep (43). Physical activity can increase total sleep time and slow-wave sleep duration while reducing rapid eye movement (REM) sleep and delaying the onset of the REM latency period (44). Furthermore, long-term exercise can enhance sleep efficiency, reduce sleep onset latency, increase total sleep duration, and promote slow-wave sleep (45, 46). Mechanisms by which exercise improves sleep include exercise-induced inflammation reduction, alterations in core body temperature, changes in the regulation of neurotransmitters involved in sleep, increases in growth hormone and brain-derived neurotrophic factor (BDNF), as well as changes in heart rate variability and autonomic nervous function (44, 45, 47–49). It is a simple, cost-effective, and non-pharmacological therapy for treating sleep disorders.

## 4 Effects of exercise therapy on sleep disorders

### 4.1 Comprehensive efficacy of exercise therapy on sleep disorders: how effective Is exercise therapy in relieving the symptoms of sleep disorder syndromes?

The evaluation of sleep disorders is fundamental to clinical diagnosis and treatment. It includes clinical assessments, subjective evaluations, and objective evaluations (6). According to ICSD-3,clinical assessments mainly encompass:

① Symptoms: Difficulty in falling asleep, frequency and duration of nighttime awakenings, early morning awakening sleep disorders, and non-restorative sleep (meaning sleep of poor quality that does not sufficiently refresh and rejuvenate);

② Duration: Days, weeks, months, years;

③ Severity: Frequency, intensity, and impact on daytime functioning (fatigue and drowsiness, cognitive function; mood disorders);

④ Psychiatric history and examination.

**Figure 1:**
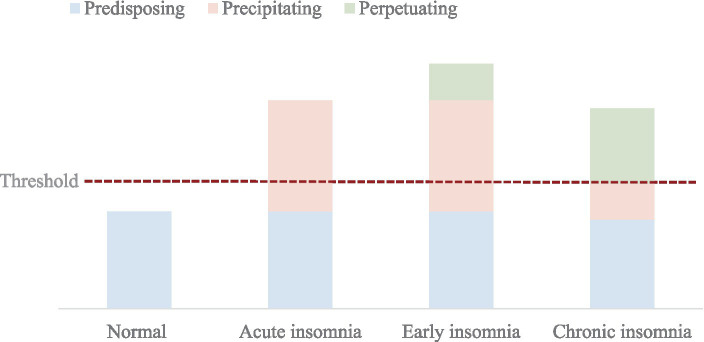
The three-factor model of sleep disorders (32): predisposing factors: these refer to an individual’s vulnerability or predisposition to developing insomnia. They include age, sex, heightened arousal, an anxious personality type, familial or personal history of sleep disorders, and genetic factors such as the presence of the short allele of the serotonin transporter gene. Precipitating factors: these are the specific triggers that lead to the onset of insomnia. Research has identified stress as one of the key risk factors in the development of sleep disorders (51, 52). Individuals are more likely to experience sleep disorders when faced with life stressors and strains, such as illness, separation, or prolonged occupational pressures. Perpetuating factors: these are the psychological and behavioral elements that allow the symptoms of insomnia to persist or even exacerbate. Factors such as irregular sleep habits (like extended time in bed) and fears regarding insomnia play a role in maintaining and prolonging the existence of sleep disorders over time. Threshold: equivalent to a dividing line for each of the 3 factors in this, varies from person to person and from time to time, but any time one of them exceeds the threshold, it can lead to sleep disorders. Acute insomnia: a form ofnon-organic insomnia that lasts less than a month. Early insomnia: difficulty falling asleep or easy awakening in the context of shorter, poorer quality sleep, usually of shorter duration and with less impact on daily life. Chronic insomnia: sleep disorders need to occur at least 3 nights per week for at least 3 months.

Subjective evaluation tools include sleep logs or diaries (for at least 2 weeks), scale evaluations (such as the Pittsburgh Sleep Quality Index (PSQI), Sleep Disorder Rating (SDRS), Epworth Sleepiness Scale (ESS), Insomnia Severity Index (ISI), Morningness-Eveningness Questionnaire (MEQ), Dysfunctional Beliefs and Attitudes about Sleep (DBAS), and FIRST, etc.). Objective measurement tools like the polysomnogram (PSG) are only used in suspected cases of sleep apnea, movement disorders, or sleep anomalies. Aurea et al. (50) randomized 20 patients with sleep disorders into resistance exercise and stretching exercise groups. The aim was to evaluate the impact of resistance and stretching exercises on sleep, mood, and quality of life in patients with chronic insomnia. After 4 months of treatment, both experimental groups showed improvements in the severity index of sleep disorders, sleep latency, awakenings after sleep onset, sleep efficiency, PSQI scores, and total sleep duration compared to the control group. Additionally, the stretching group exhibited lower levels of tension and anxiety than the control group. The study results indicated that both therapeutic methods could effectively alleviate the symptoms of sleep disorders, improving objective sleep, emotional state, and quality of life. Yue et al. (13) included 30 middle-aged women with sleep disorders in their study. They designed an intervention focused on brisk walking. After a 2-month exercise intervention, 99% of the participants reported significant improvements in sleep quality. The proportion of participants achieving over 6 h of sleep rose from 13% before the intervention to 82.5% afterward. Symptoms such as shallow sleep, frequent awakenings, dream-filled sleep; waking up feeling tired, foggy-headed, with headaches or dizziness; and anxiety, irritability, tension, worry, suspicion, depression, etc., caused by sleep disorders were all mitigated (Figure 1).

### 4.2 Improvement of sleep quality through exercise therapy: how does exercise therapy improve sleep quality?

**Figure 2:**
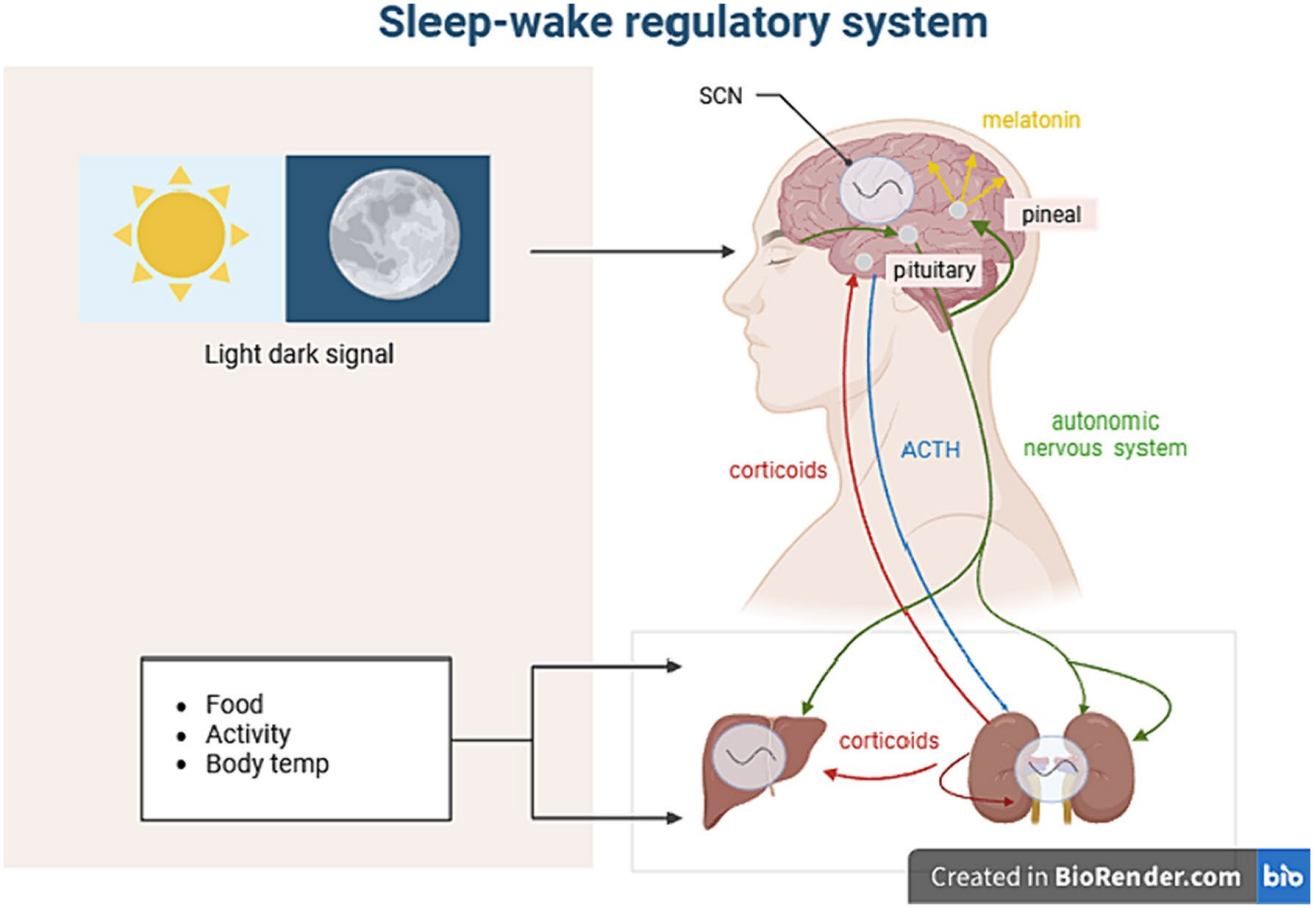
The brain’s circadian rhythm regulator is located within the Suprachiasmatic Nucleus (SCN) and is greatly influenced by light and external environments. The intrinsically photosensitive retinal ganglion cells (ipRGCs) are responsible for receiving signals and sending them to the SCN. The SCN integrates and coordinates external input rhythms (such as sleep–wake cycles, eating-feeding cycles, and activity-rest cycles) to control the balance of bodily states, including heart rate, body temperature, and hormone levels. Melatonin is a light signaling hormone secreted by the pineal gland, and its biosynthesis is closely linked to the photoperiod. Melatonin mainly regulates the sleep phase, and the level of melatonin at night directly affects the quality of sleep. Reduced melatonin levels are one of the most important signs of sleep disorders and a series of dysfunctions.

The Pittsburgh Sleep Quality Index (PSQI) (53), formulated by Dr. Buysse and colleagues from the Psychiatry Department of the University of Pittsburgh in 1989, is the most commonly used method for assessing sleep quality both clinically and in research. It is suitable for assessing sleep quality in patients with sleep disorders, those with psychiatric disorders, as well as the general population. PSQI consists of seven domains and 23 items: subjective sleep quality, sleep latency, sleep efficiency, sleep disorders, use of sleep medication, and daytime dysfunction. The cumulative score is known as the PSQI total score (ranging from 0 to 21), with higher scores indicating poorer sleep quality (Figure 2).

Dadgostar et al. (54) included 32 patients with sleep disorders to study the effects of exercise on the symptoms of sleep disorders. 16 of them underwent combined resistance-aerobic exercise therapy, with the rest as the control group. The intervention group underwent a 12-week program, doing aerobic exercise for 3 days a week and resistance training for the other 3 days. The patients’ sleep quality was assessed using the PSQI before and after the intervention. The results showed that combined aerobic-resistance exercise could improve sleep quality in patients with sleep disorders, enhance subjective sleep quality and actual sleep duration, and reduce daytime dysfunction caused by sleep issues. The findings support the effectiveness of exercise therapy on sleep quality, suggesting that organized physical activity combining aerobic and resistance exercises can improve sleep quality. Banno et al. (55) conducted a systematic review and meta-analysis to study the efficacy of exercise on patients with sleep disorders. Their study included nine randomized controlled trials with a total of 557 participants. The impact of exercise on various sleep parameters for patients with sleep disorders was measured and analyzed using a random effects model for the meta-analysis. Compared to the control group, the intervention group’s PSQI score averaged 2.87 points lower, indicating the effectiveness of exercise therapy in improving sleep quality. Zhu Jinya et al. (56) provided exercise interventions like the Eight-Section Brocades (a form of Qigong) and broadcast gymnastics to 48 patients with chronic schizophrenia accompanied by sleep disorders. They found that the intervention group had significantly better sleep quality, sleep duration, sleep efficiency, fewer sleep disorders, and a lower PSQI total score compared to the control group, suggesting that the combination of Eight-Section Brocades and broadcast gymnastics can effectively improve sleep status and quality of life for patients with schizophrenia. Cristini et al. (57) conducted a systematic review through meta-analysis to determine the evidence supporting the improvement of sleep quality in Parkinson’s Disease (PD) patients through exercise. The research data came from 10 randomized controlled trials and two non-randomized controlled trials, involving a total of 690 PD patients. The data indicated a significant positive effect of exercise on sleep quality, underscoring the potential of exercise to improve sleep quality in PD patients.

### 4.3 The efficacy of exercise therapy on sleep duration

Currently, the methods for assessing sleep duration are mainly divided into subjective sleep measurements and objective sleep measurements. Subjective sleep measurements are based on survey methods (primarily retrospective questions). The most common questions involve asking about sleep duration in one night, such as “How many hours do you sleep in one night over the past 4 weeks (58)?” or “How many hours do you usually sleep in one night over the past 2 weeks (59)?.” Some studies inquire about usual bedtime and wake-up time, then calculate the duration. This approach might be more advantageous than merely asking about total duration (60).

Objective sleep measurements are based on physiological monitoring to assess sleep duration. Presently, there are two primary objective methods: polysomnography (PSG) and actigraphy. PSG evaluates sleep based on brainwave activity and is considered by many as the “gold standard” for defining sleep (61). Actigraphs are often worn on the wrist and estimate sleep based on arm movements, utilizing multi-directional accelerometers to measure activity counts during each period. Activity counts are used to delineate periods of sleep or wakefulness. These devices can store multiple days of data and produce total duration along with other sleep parameters. Both methods aren’t suitable for population-based research. Studies have shown that the average self-estimated physiological sleep duration is between 6 and 6.5 h (60), yet sleep durations estimated by PSG or actigraphy are shorter than self-reported durations.

Most research indicates that exercise interventions can, to some extent, reduce sleep latency and the duration of awakenings after sleep onset, increasing total sleep duration. A four-week randomized controlled trial recruited 40 adults with poor sleep quality, dividing them into exercise and control groups. The exercise group underwent 6 weeks of moderate-intensity aerobic exercise, three times a week, 45 min each session. Statistical data showed that the total sleep time in the exercise group increased by an average of 24 min compared to the control group. Additionally, the REM sleep duration of the exercise group also increased. Populations that reported higher levels of physical activity also tend to have longer sleep durations (14). Khalsa et al. (62) included 34 female patients with chronic sleep disorders. Participants maintained sleep–wake diaries during a 2-week pre-treatment baseline period and an 8-week yoga intervention. During the intervention, the same practices were performed daily, with the practice time gradually increasing from 30 min to 1 h. Measurements of sleep efficiency, total sleep time, total wake time, sleep onset latency, wake time after sleep onset, number of awakenings, and sleep quality indicators all showed significant improvements compared to baseline values.

### 4.4 Application of exercise therapy in different populations

Exercise therapy has shown significant benefits in the treatment of sleep disorders in the elderly (63). Endeshaw et al. (64) analyzed data from 7,162 older adults in the US to investigate the association between social activities and physical exercise and sleep disorder symptoms in the elderly. The data revealed that the likelihood of sleep disorder symptoms significantly decreased in older adults who participated in social activities and/or walking exercises. Specifically, the probability of sleep disorder symptoms was reduced by 30 and 22% for those participating in social activities and walking exercises respectively, and 40% for those participating in both activities. Reid et al. (65) conducted a randomized controlled trial to investigate the effects of moderate aerobic exercise on sleep, mood, and quality of life in elderly patients with chronic sleep disorders. After 16 weeks of aerobic exercise, the group who participated in physical exercise showed improvements in overall PSQI scores, sleep latency, sleep duration, daytime dysfunction, and sleep efficiency PSQI scores. Additionally, symptoms of depression and daytime sleepiness were also reduced. Li Chao et al. (66) recruited 150 elderly patients with sleep disorders from the community and randomly divided them into a control group and a Tai Chi exercise group (exercising daily, at least 5 days a week). The efficacy of exercise treatment for sleep disorder symptoms in the elderly was observed over 3 months using the SPIEGEL score as the efficacy evaluation criterion. Compared to the control group, the Tai Chi exercise group showed a significantly better treatment effect. Bonardi et al. (67) investigated the effects of different types of physical exercises on the sleep quality of older adults. Participants aged 60–75 were randomly divided into three groups: an aerobic group, a combined aerobic and resistance group, and a control group. Training lasted for 10 weeks with 30 continuous sessions. Sleep activity was continuously monitored using an activity recorder. Compared to the control group, the aerobic group and the combined aerobic and resistance group showed reduced sleep fragmentation indices, increased sleep efficiency, decreased total activity scores during sleep, and increased percentages of still time, indicating improved sleep quality. The results suggest that regular moderate exercise can improve sleep quality in the elderly, regardless of the type of physical activity. Bademli et al. (68) conducted a study involving 60 older adults with mild cognitive impairment, dividing them into an experimental group and a control group, each comprising 30 participants. The experimental group underwent a 20-week “sports activity program” that included a 10-min warm-up as the initial phase, 20 min of rhythmic exercises as the active phase, a 10-min cooldown as the final phase, and 40 min of free walking. The average SMMSE (Mini-Mental State Examination) score for the experimental group was higher than that for the control group, and the average PSQI score increased significantly compared to before the intervention. This study indicates that the physical exercise program improved both cognitive function and sleep quality in the elderly.

Given the physiological characteristics of adolescents, exercise therapy holds significant potential for addressing sleep disorders in this age group (69). Mendelson et al. (70) examined the effects of exercise training on improving sleep duration, sleep quality, and physical activity in obese adolescents. 20 obese adolescents participated in a 12-week combined aerobic and resistance training program, including 180 min of exercise per week. After the training, there was an increase in sleep duration and efficiency among the obese adolescents. The study confirmed that exercise training could enhance sleep duration, quality, and physical activity in obese adolescents. Another study on 51 adolescents over 3 weeks, which included 30 min of moderate-intensity exercise every weekday morning, showed that compared to the control group (non-exercising group), there were improvements in objective sleep duration and sleep efficiency, with reduced sleep time and increased REM sleep latency (71). Jiang Ming et al. (72) selected 360 university students, dividing them into severe sleep disorder (20 participants), moderate sleep disorder (25 participants), mild sleep disorder (50 participants), and an equivalent number of non-sleep-disordered participants as a control group. They explored the effect of exercise therapy on improving sleep disorder status in university students. After 4–6 weeks of exercise intervention, the improvement rates in the severe, frequent, and occasional sleep disorder groups were statistically significant compared to the control group (*p* < 0.01). The research data indicate that a reasonable sports exercise program can effectively ameliorate sleep disorders in university students (Table 1).

**Table 1:** Classification and symptoms of sleep disorders (28).

Classification	Diagnosis	Symptoms
Insomnia	Chronic insomnia	Difficulty in falling asleep and maintaining sleep
Sleep-related breathing disorders	Obstructive sleep apnea syndrome (OSAS)	Excessive daytime sleepiness
Central disorders of hypersomnolence	Narcolepsy	Excessive daytime sleepiness, cataplexy (associated with narcolepsy)
Circadian rhythm sleep–wake disorders	Shift work, Jet lag syndrome	Difficulty in sleep initiation and maintenance, early awakening, excessive daytime sleepiness, indigestion
Parasomnias	Somnambulism (Sleepwalking)	Getting out of bed and walking around while asleep
Sleep-related movement disorders	Restless legs syndrome	Urge to move the legs, difficulty falling asleep
Independent syndromes	Normal variants and undefined problems	
Other sleep disorders		

### 4.5 Formulation of exercise therapy protocols

The influence of exercise on sleep is modulated by individual characteristics and specific exercise programs. Individual attributes encompass sex, age, health status, type of sleeper, and Body Mass Index (BMI). In contrast, exercise protocols might vary based on their acuteness or regularity, aerobic or anaerobic nature, as well as their intensity, duration, environment (indoor or outdoor, hot or cold conditions), and the time of day they are performed (73).

In exercise intervention programs for sleep disorders, the types of exercise primarily include: moderate-intensity aerobic exercises (such as running, cycling), mind–body exercises (like yoga, Tai Chi, Ba Duan Jin, and Pilates), resistance training, and daily walking. Exercise intensity refers to the degree of effort exerted by the body during a physical activity. It describes how easy or challenging an activity is, in relation to an individual’s current maximal capability. The relative intensity of aerobic exercises can be described using the percentage of one’s aerobic capacity (VO2max), percentage of maximum heart rate, or other similar measurements. Alternatively, it can also be gauged by an individual’s perceived exertion levels for an activity: very light, light, moderate, hard, very hard, or maximum (74). For commonly used methods to determine aerobic exercise intensity, please refer to Table 2.

**Table 2:** Commonly used methods to determine aerobic exercise intensity (75).

Intensity level	%HRR, %VO2R	%HRmax	%VO2max	RPE (0–10)	Talk test
Low	<30	<57	<37	Very easy (<3)	Can talk and sing
Somewhat low	30 ~ 39	57 ~ 63	37 ~ 45	Very easy to easy (3 ~ 4)	
Moderate	40 ~ 59	64 ~ 76	46 ~ 63	Easy to somewhat hard (5 ~ 6)	Can talk but cannot sing
High	60 ~ 89	77 ~ 95	64 ~ 90	Somewhat hard to hard (7 ~ 8)	Cannot speak in complete sentences
Near max to max	≥90	≥96	≥91	Very hard (≥9)	

HRR = heart rate reserve; VO2R = oxygen uptake reserve; HRmax = maximum heart rate; VO2max = maximum oxygen uptake; RPE, rating of perceived exertion scale.

Research suggests that moderate aerobic exercise has a positive impact on sleep quality, with the greatest effect size for improving sleep disorders observed in medium-intensity aerobic exercises (76). In contrast, low physical activity exercises (like yoga) did not demonstrate significant effects (77). A randomized controlled trial by Kjeldsen et al., published in Bioenergetics 2012, assessed the dose–response effect of aerobic exercise on sleep duration, efficiency, and quality among sedentary, moderately overweight men. The study enrolled 53 sedentary white males aged 20 to 40, participating in a 13-week aerobic exercise intervention. Sleep duration was objectively measured over 3 days using an activity monitor, revealing that the high-dose group (performing physical activity consuming 600 kcal/day) showed a significant increase in sleep duration. The results indicated that daily high-dose aerobic exercise for 13 weeks can extend sleep duration. Passos et al. (11) evaluated and compared the acute effects of three different forms of physical exercise on the sleep patterns of patients with chronic primary insomnia. They divided 48 patients with sleep disorders into four groups: a control group of 12, a high-intensity aerobic exercise group of 12, a medium-intensity aerobic exercise group of 12, and a medium-intensity resistance exercise group of 12. Polysomnography data revealed that the medium-intensity aerobic exercise group had an increased total sleep time, confirming that medium-intensity aerobic exercise can improve sleep in patients with chronic primary insomnia.

The appropriate duration and frequency of exercise (where frequency refers to the number of days per week the exercise plan is executed) are crucial factors affecting sleep quality. The World Health Organization recommends, for the promotion and maintenance of health, a minimum of 5 days per week of medium-intensity aerobic physical activity for 30 min each time, or a minimum of 20 min of high-intensity aerobic exercise 3 days a week (74). Gong Mingjun et al. (76) used meta-analysis research methods, incorporating a sample of 1,270 subjects. They employed a random-effects model for overall effect size and heterogeneity tests to examine the effects of exercise interventions on sleep disorders. Intervention durations ranged from 4 to 48 weeks, divided into groups of 4–9 weeks, 12 weeks, 16–18 weeks, 24–25 weeks, and 48 weeks. Single exercise durations varied from 30 to 90 min, categorized into groups of 30–50 min, 55–60 min, and 70–90 min. Exercise frequency ranged from 2–7 times per week, divided into groups of 2 times/week, 3 times/week, and 4–7 times/week. Data analysis revealed that a regimen of exercising about 60 min each session, 3 times a week, for approximately 16 weeks had the most significant effect in improving sleep disorders. However, other research suggests that shorter exercise training durations (like 16 weeks of medium-intensity exercise) only influence the subjective sleep quality assessed by the PSQI and overall sleep score evaluations, with no impact on objective sleep measures (73).

Exercise therapy encompasses a wide range of content, and patients receiving exercise interventions often have one or several other diseases or functional impairments. This can increase the risk factors during exercise therapy. Furthermore, improper or excessive exercise can also lead to harm, such as cardiovascular events during the activity and the risk of exercise-induced injuries (75). To reduce exercise-related risks and design targeted exercise intervention plans, the Chinese Expert Consensus on Exercise Prescription (75) formulated the basic precautions for exercise intervention programs:

1. Individuals receiving exercise interventions should undergo fitness assessments. The main indicators (75) include:
2. Cardiopulmonary endurance (observing cardiovascular responses during exercises with various loads).
3. Body composition or Body Mass Index (BMI).
4. Muscular strength and endurance.
5. Flexibility.
6. Balance capability. Based on the level of fitness and health condition, the exercise intensity should be determined.
7. A warm-up or preparatory activity should be performed before the exercise, and cooling down and stretching exercises should be conducted after the activity. For instance, some exercise intervention studies typically involve 10-min warm-ups and cool-down stretching exercises both before and after the main activity (54).
8. During the exercise, close attention should be paid to the participants’ complaints and any changes in their vital signs. If symptoms such as pallor, nausea, vomiting, and dizziness occur, exercise should be halted immediately, and a physician should be notified for symptomatic treatment (56).
9. Special attention and monitoring should be intensified for specific populations, including pregnant women, the elderly, children and adolescents, children with disabilities, and patients on medication.

## 5 Comparison of exercise therapy with other treatment methods

### 5.1 Comparison between exercise therapy and pharmacological treatment

Sleep disorders can be treated with non-pharmacological and pharmacological approaches. There are a variety of medications for treating sleep disorders, including over-the-counter drugs (OTCs; antihistamines, melatonin, and herbal preparations) and prescription drugs (BzRAs, chronobiotics, low-dose doxepin), as well as other drugs not specifically for sleep disorders (antidepressants, antipsychotics, anticonvulsants). Antihistamines can cause drowsiness; side effects include agitation, anticholinergic effects (such as dry mouth, urinary retention), and worsening restless legs syndrome. Tolerance can develop, and sudden cessation can lead to rebound insomnia (7). Melatonin is a hormone produced by the pineal gland that helps reinforce circadian and seasonal rhythms. The efficacy of melatonin as a sleep aid remains inconclusive; a meta-analysis showed that sleep latency was reduced only by 7.2 min (78). However, using melatonin might cause side effects like drowsiness, dizziness, headache, and nausea. Herbal preparation valerian improves responses to GABA by binding to the GABA A receptor (79). A meta-analysis found that valerian improved subjective sleep quality, although quantitative measures of sleep were unchanged (80). Its side effects include drowsiness, dizziness, and allergic reactions.

Chunling et al. (81) recruited 100 patients with primary sleep disorders and divided them randomly into a study group and a control group. The study group received aerobic exercise combined with zopiclone treatment, while the control group was treated with zopiclone alone. After 8 weeks of intervention, the PSQI and polysomnography (PSG) were used to assess sleep quality and structure. The data showed significant differences in sleep quality improvement indices between the two groups, suggesting that aerobic exercise combined with medication intervention had superior effects in improving sleep quality and structure compared to medication alone. Another study by Daiqu et al. (82) researched the long-term effects of yoga training on patients with chronic sleep disorders. Out of 200 patients, 100 were in the control group and 100 in the observation group. Both groups received pharmacological treatment, but the observation group also practiced yoga. At the end of the drug treatment, both groups showed significant improvements in sleep quality with no significant statistical difference. However, at the 12-week follow-up, the observation group’s therapy outcomes were superior to the control group’s. It was found that combining pharmacotherapy with cognitive behavioral therapy could offer additional benefits. Using drugs to achieve short-term results, then gradually reducing and finally stopping the drug dosage while persisting with cognitive behavioral therapy can yield notably stable long-term effects (5, 83). However, prolonged usage of sleep medications might lead to dependencies. Especially in older populations, medication can increase body fat, reduce total body water and plasma proteins, leading to an increased half-life of the drug’s elimination and potential adverse reaction risks. Thus, non-pharmacological treatments should be prioritized in the elderly before considering drugs (84, 85).

In conclusion, pharmacotherapy offers a variety of drug choices and is recognized in both domestic and international guidelines for improving sleep disorder symptoms, like benzodiazepines having both sedative and anti-anxiety effects. However, risks such as potential drug dependency, drug tolerance, or cognitive impairments exist, and abrupt cessation can result in withdrawal symptoms. In contrast, exercise therapy is healthy, safe, easily mastered, and cost-effective. Although it may take longer to see effects, its efficacy tends to gradually strengthen and lasts longer. Considering the efficacy and safety of exercise in treating insomnia, it’s recommended that clinicians might prioritize exercise therapy for sleep disorder patients who experience side effects from sedative-hypnotic drugs.

### 5.2 Comparison of exercise therapy and other cognitive behavioral therapies

Cognitive Behavioral Therapy for Insomnia (CBT-I) encompasses psychological and behavioral protocols such as sleep restriction, stimulus control, relaxation, cognitive strategies, and sleep hygiene education. The goal is to modify factors leading to sleep disorders, which include behavioral factors (like poor sleep habits and irregular sleeping schedules), psychological factors (such as unrealistic expectations, worries, and unproductive beliefs), and physiological factors (mental and physical tension, hyperarousal). Its primary indications are chronic sleep disorders, either primary or comorbid, as well as sleep disorders among both the young and elderly. Major challenges faced by CBT-I in implementation include: ① Difficulties in clinical use due to lack of patient adherence; ② Limited number of practitioners trained in CBT-I, with most not operating within medical settings (86). Unlike traditional psychological interventions, exercise therapy relies less on highly trained and specialized clinicians for implementation.

A randomized controlled trial (87) randomly assigned 37 insomnia patients to either a zero-time exercise group (a type of exercise integrated with lifestyle) or a sleep hygiene education group. The insomnia severity index was significantly lower in the zero-time exercise group than in the sleep hygiene education group. Simple and brief exercise training demonstrated high acceptability and exercise adherence, proving effective in alleviating the severity of sleep disorders. Yu et al. (88)compared the long-term effects of exercise therapy, CBT-I, and pharmacotherapy on improving sleep in adult patients with chronic insomnia. Their findings suggested that both exercise and CBT-I manifested better long-term effects in improving sleep, while pharmacological interventions were less effective in the long run. Another study (89) explored the comparative effectiveness of CBT-I, Tai Chi, and sleep education control on primary outcomes of insomnia diagnosis and secondary outcomes of sleep quality, fatigue, depressive symptoms, and inflammation in elderly patients with insomnia. CBT-I showed superior efficacy in alleviating clinical sleep disorders compared to Tai Chi and sleep education control groups (*p* < 0.01). It also demonstrated larger and more sustained improvements in sleep quality, sleep parameters, fatigue, and depressive symptoms. However, when compared to the sleep education control group, the Tai Chi group showed improvement related to sleep quality, fatigue, and depressive symptoms (all *p* < 0.05), but not in relieving insomnia. This study does not support the use of Tai Chi as a treatment method for significant sleep disorders in the elderly, suggesting that cognitive behavioral therapy can better achieve and maintain the treatment of sleep disorders in later life.

In summary, current comparative studies between exercise therapy and CBT-I are relatively few, and the conclusions drawn from the limited research are not consistent. Although CBT-I is recommended as the first-line treatment for sleep disorders by the American Academy of Sleep Medicine and various authoritative official organizations, its accessibility is limited due to the need for well-trained therapists and high treatment costs. This restricts the reach and scalability of CBT-I in the community. Given that there is no significant difference in the long-term efficacy between exercise therapy and CBT-I, and considering the low cost and higher accessibility of exercise therapy, it is recommended as an adjunctive therapeutic approach for the long-term management of chronic sleep disorders in adults.

## 6 Potential mechanisms of exercise therapy in improving sleep disorders

### 6.1 Effects of exercise on the sleep regulatory system

The existence of sleep regulation mechanisms has been confirmed (90). Some of the influencing factors include growth hormone-releasing hormone (GHR), cytokines, interleukin-1, prostaglandin D2, adenosine, tumor necrosis factor (TNF), prolactin 100, adrenocorticotropic hormone-related peptides, and vasoactive intestinal peptide-related peptides (91). Acute exercise has been shown to increase cytokines, which in turn are related to sleep regulation. All these factors play a role in sleep restoration. In particular, inflammatory factors not only participate in inflammation and immune response but also serve as the pivotal factors for the neural-endocrine-immune network liaison, participating in the regulation of sleep–wake rhythms (81). Short-term sleep disorders activate the body’s stress response, inducing chronic inflammatory responses. Inflammatory factors (TNF-α, IL-1β, IL-6) moderately rise, specifically affecting NREM sleep and increasing SWS (92). Long-term sleep disorders can significantly increase the blood levels of inflammatory factors. Excessively high inflammatory factors not only fail to promote sleep but also induce the central nervous system to produce neurotoxic substances, exacerbating sleep disorder symptoms (93, 94). Therefore, altering the body’s levels of inflammatory factors plays a vital role in regulating sleep. There is a close relationship between exercise, inflammatory factors, and sleep, where exercise can improve many pro-inflammatory factors.

It is reported that moderate exercise training can also promote immune function (95). Symptoms of depression, poor sleep quality, and systemic inflammation markers (like IL-6) are often associated with this (96). Researchers have found that an increase in wake time during the first non-rapid eye movement (NREM) sleep cycle is negatively correlated with the count of natural killer (NK) cells (97). Compared to patients with sleep disorders, those with good sleep have significantly higher levels of CD3+, CD4+, and CD8+ cells in the body (98). Although the improvement of immune function is definitely related to sleep, it is still unclear whether this is a cause or a result. Research by Chunling et al. (81) showed that after aerobic exercise intervention, the experimental group’s inflammatory factors TNFα, IL-1β, and IL-6 levels were lower than the control group. They speculated that the mechanism of aerobic exercise promoting sleep might be through adjusting the imbalance of inflammatory factors after sleep loss, thereby changing the sleep structure and reconstructing a stable sleep–wake rhythm. Meta-analysis research on the impact of acute exercise on sleep structure showed slight increases in slow-wave sleep (SWS) and rapid eye movement (REM) sleep latency, while the duration of REM sleep decreased (99, 100). In elderly rats, 8 weeks of chronic aerobic exercise starting at night (equivalent to human morning) significantly reduced sleep fragmentation (by 35%) during non-rapid eye movement sleep and increased EEG Delta wave power. After exercise cessation, the sleep changes persisted for 2 weeks, indicating a lasting impact of exercise on the central nervous system (101).

The above findings suggest that exercise may have an impact on sleep by modulating inflammatory factors and participating in the neuro-endocrine-immune network regulatory system.

### 6.2 Exercise and its adjustment of the biological clock

The circadian rhythm, also known as the biological clock, is an endogenous rhythm of life activities that operates on a 24-h cycle. It responds to endogenous factors such as core body temperature and exogenous factors like light. Any disturbances to these factors can result in rhythm disorders, thereby affecting sleep. Exercise, as a behavioral countermeasure promoting the adaptation of circadian rhythms, has been studied. Youngsted et al. (90) established phase response curves, recording the magnitude and direction of phase shifts related to the circadian timing of exercise. The study involved 51 elderly and 48 young individuals, subjecting them to circadian rhythm measurements for up to 5.5 days, and one-hour of moderate treadmill exercise at one of the eight times during the day/night across three consecutive days. The results indicated a pronounced phase delay between 7:00 to 10:00 and a significant phase advance between 7:00 and 1:00 to 4:00, suggesting that exercise can regulate the phase timing of the human circadian system. Barger et al. (102) recruited 18 healthy young males to participate in a 15-day randomized clinical trial. After delaying the sleep–wake time by 9 h, they conducted a week of nighttime exercise or non-exercise control, measuring circadian rhythms before and after the exercise. The study data showed that exercise can significantly delay the phase of the human circadian pacemaker and may help promote circadian adaptation to schedules requiring a delay in the sleep–wake cycle.

### 6.3 Impact of exercise on mental health

Studies have shown a relationship between exercise and improvements in mental health, including mood states and self-esteem (103). For instance, yoga can achieve mind–body integration through postures, breath training, and guided mindfulness meditation. It has the capability to stabilize the autonomic nervous system, alleviate stress, and eliminate mental tension. It indirectly massages and stimulates certain glands, resulting in psychological stability, thus improving sleep, reducing anxiety and depressive moods, and breaking the vicious cycle of negative emotions leading to sleep disorders (103). Sobana et al. (104) selected 40 male patients with sleep disorders, divided randomly into experimental and control groups. The experimental group underwent 8 weeks of yoga treatment (yoga sessions in the morning, with each session lasting 90 min). There was a significant improvement in stress scores and self-confidence scores. Regarding acute exercise, research indicates that 20–40 min of aerobic exercise can sustainably improve anxiety states and mood for several hours (105, 106). The neurobiological mechanism hypothesis suggests that participation in sports activities enhances cognition and mental health by altering the brain’s structure and functional composition. Increasing energy expenditure through activities might influence sleep patterns, subsequently improving mental health outcomes (107). It is speculated that exercise increases energy expenditure and body temperature, thus promoting sleep to recover the body. A “trigger” for the onset of sleep is the evening drop in body temperature, primarily mediated by an increase in peripheral skin blood flow. Exercise causes core temperature to rise, activating the hypothalamus-controlled heat dissipation mechanism, thus promoting sleep onset. Sleep disorder patients often exhibit impaired nocturnal temperature down-regulation. Studies in rodents indicate that running can increase cell proliferation, survival, and differentiation (108). Additionally, exercise stimulates the growth of new capillaries, essential for delivering nutrients to neurons (109). Neurochemicals such as brain-derived neurotrophic factor, insulin-like growth factor 1, and vascular endothelial growth factor are highly concentrated in the hippocampus and other brain areas. These neurochemicals increase with exercise and promote the downstream effects of aerobic exercise on brain structure, function, and cognition. Physical activity impacts health via the release of endorphins and their interaction with other neurotransmitter systems. It remains unclear whether the short-term pleasure individuals experience during physical activity is due to endorphins and to what extent this activity contributes to improved mental health over time.

There is a link between serotonin and mood, where serotonin helps alleviate anxiety and depression and regulate mood. Chronic sleep disorders might result from decreased serotonin activity, and exercise (running) positively affects free tryptophan in rat serum (38). Chaouloff et al. (110) found that intense exercise (running) increases the brain’s serotonin (5-hydroxytryptamine, 5-HT) synthesis in two ways: (1) hydrolysis-induced release of free fatty acids in the blood replaces the binding of the essential amino acid tryptophan with albumin, increasing free tryptophan concentration, and (2) the increased ratio of circulating free tryptophan to amino acids competing with tryptophan at the blood–brain barrier leads to more tryptophan entering the brain. This suggests that exercise enhances a feedback regulation mechanism. Indirect indicators of 5-HT function reveal the likelihood associated with the increase in 5-HT biosynthesis induced by acute exercise and/or the increase in 5-HT release.

## 7 Conclusion and future directions

Based on the aforementioned retrospective research findings, we can tentatively conclude that exercise therapy demonstrates potential positive effects in alleviating sleep disorders. Exercise therapy can not only mitigate the overall symptoms of sleep disorders but also improve sleep quality and prolong sleep duration sleep disorders. Some preliminary studies on molecular mechanisms have provided clues, suggesting that exercise therapy might exert its therapeutic effects by modulating neurotransmitters, hormones, and sleep regulatory systems. Beyond its impacts on sleep, exercise has also been proven to offer numerous other benefits to human health, including but not limited to enhancing physical and mental well-being and improving the quality of life for individuals with chronic diseases. Given its low cost, accessibility, capability to be performed individually or in groups, exercise therapy stands out as a promising alternative treatment.

Despite these positive findings, several issues still need further exploration and resolution. The design of current exercise intervention programs lacks standardized reference indices and criteria, leading to variations in therapeutic effects among different individuals. There is a dearth of studies in the current literature that delve into the mediating variables between exercise interventions and sleep disorders, such as the content of the exercise intervention, total duration of the intervention, frequency, duration of each session, form of practice, and timing of the practice. These components are essential when drafting a plan for exercise interventions for sleep disorders, and the absence of related studies directly results in the current exercise plans falling short in offering guiding recommendations for sleep disorders. Thus, future research could delve deeper into the optimal types, intensities, frequencies, durations, practice locations, control group activities, sex, and different age groups, among other modulating variables, for exercise therapy. Such endeavors will ensure that intervention strategies for sleep disorders are more precise and effective.

## Author contributions

YZ: Writing – review & editing, Writing – original draft, Data curation. CL: Writing – review & editing, Writing – original draft, Conceptualization. QD: Data curation, Investigation, Writing – original draft. YL: Data curation, Investigation, Writing – review & editing.
